# Long-Term Incubation PrP^CWD^ with Soils Affects Prion Recovery but Not Infectivity

**DOI:** 10.3390/pathogens9040311

**Published:** 2020-04-23

**Authors:** Alsu Kuznetsova, Debbie McKenzie, Catherine Cullingham, Judd M. Aiken

**Affiliations:** 1 Agricultural, Life and Environmental Sciences Faculty, University of Alberta, Edmonton, AB T6G 2G8, Canada; alsu@ualberta.ca; 2 Faculty of Science, University of Alberta, Edmonton, AB T6G 2M8, Canada; debbie.mckenzie@ualberta.ca; 3 Department of Biology, Carleton University, Ottawa, ON K1S 5B6, Canada; catherine.cullingham@carleton.ca

**Keywords:** prion protein, soil, CWD, prolonged incubation, CWD infectivity, prion detection

## Abstract

Chronic wasting disease (CWD) is a contagious prion disease of cervids. The infectious agent is shed from animals at the preclinical and clinical stages of disease where it persists in the environment as a reservoir of CWD infectivity. In this study, we demonstrate that long-term incubation of CWD prions (generated from tg-mice infected with deer or elk prions) with illite, montmorillonite (Mte) and whole soils results in decreased recovery of PrP^CWD^, suggesting that binding becomes more avid and irreversible with time. This continual decline of immunoblot PrP^CWD^ detection did not correlate with prion infectivity levels. Bioassay showed no significant differences in incubation periods between mice inoculated with 1% CWD brain homogenate (BH) and with the CWD-BH pre-incubated with quartz or Luvisolic Ae horizon for 1 or 30 weeks. After 55 weeks incubation with Chernozem and Luvisol, bound PrP^CWD^ was not detectable by immunoblotting but remained infectious. This study shows that although recovery of PrP^CWD^ bound to soil minerals and whole soils with time become more difficult, prion infectivity is not significantly altered. Detection of prions in soil is, therefore, not only affected by soil type but also by length of time of the prion–soil interaction.

## 1. Introduction

Chronic wasting disease (CWD), a contagious prion disease affecting different cervid species, has been present in the United States for at least 50 years. Initially identified in the western U.S., the disease has spread through much of western and central North America. The disease has also been confirmed in Korean and European cervids [1]. A significant challenge for disease management and surveillance is that infectious CWD prions are shed during both the preclinical and clinical stages of disease [2,3,4]. The shed prions can remain infectious even after prolonged periods in the environment [5,6,7]. Upper soil horizons can serve as an environmental reservoir for prions [8,9,10,11,12], and, therefore, facilitate horizontal transmission of CWD [13,14]. 

Soil properties are a key factor for CWD persistence and transmission in the environment [8,15,16]. Whereas the soil mineral montmorillonite (Mte) avidly binds abnormal prion protein, enhancing infectivity [17,18], an organic soil constituent, humic acids can decrease CWD infectivity [19]. Prion persistence under harsh environmental conditions has been described [20,21], demonstrating prions shed in native soil maintain infectivity for years [6,22]. In addition, laboratory soil column experiments indicated that PrP^Sc^ was stable after incubation in the soil for 18 months [23], and soil-bound hamster prions provided a catalytically active seed for protein misfolding cyclic amplification (PMCA) after a year-long incubation [24]. These data suggest that soil is likely to be an important environmental reservoir of prion infectivity. One study demonstrated the presence of PrP^Sc^ in naturally contaminated soils [12]. A number of laboratory studies have, however, suggested a time-dependent decline in the ability to detect rodent prions when bound to soils [22,25]. A decline in recoverable PrP^Sc^, after prolonged incubation, was found to be soil type specific with less PrP^Sc^ recovery from clay compared to sandy soils [23].

Sensitive detection of infectious CWD prions from environmental samples would have clear benefits for monitoring CWD spread. We demonstrate that, with time, PrP^CWD^ becomes more difficult to recover from variety of soil types, yet CWD infectivity does not significantly decrease. Our findings suggest significant limitations to soil-bound PrP^CWD^ detection especially for soils with montmorillonite minerology, impacting both surveillance and mitigation approaches.

## 2. Materials and Methods

### 2.1. Soils and Minerals

We studied eight surface soil horizons from 7 different soils: horizons LFH (plant litter horizon) and Ae (illite-enriched mineral horizon) of Luvisol, horizons LFH (plant litter) and Bf (illite- and iron-enriched mineral horizon) of Brunisol, Ah (humic) horizons of two Chernozemic soils, and Ae (quartz–illite mineral horizon) of two Podzolic soils. All soil samples (except Podzols) were collected in Canada and represent soil cover of boreal, tundra and prairie regions. Podzols, Luvisols and Brunisols are found in boreal and tundra ecozones, while the Chernozems are dominant in the prairies [8]. Properties of the soils are summarized in Kuznetsova et al. [24]. Minerals used for binding experiments were purchased from Ward’s Science: quartz (#940005), illite (#46E0315) and montmorillonite (Mte, #46E0435).

### 2.2. Ethics Statement 

All work with animals was performed in compliance with the Canadian Council on Animal Care Guidelines and Policies. All procedures involving animals were reviewed and approved by the Health Sciences Animal Care and Use Committee of the University of Alberta.

### 2.3. Prion Preparation, Binding Experiments and Immunoblotting

CWD-infected brain homogenates were prepared from transgenic mice expressing either 132MM elk PrP (tgElk) or wild-type white-tailed deer PrP (tg33) infected with elk or white-tailed deer prions. Uninfected brain homogenates were prepared from non-infected tgElk or tg33 mice. Brain tissues were homogenized (10% *w*/*v*) in water and, for some experiments, clarified at 800 g for 5 min [11]. In binding experiments, identical amounts of 10% brain homogenate (BH^CWD^ or uninfected NBH; final concentration 0.25%) were incubated with water (control) or a binding agent (solid phase minerals or soil horizons) in silanized Eppendorf tubes at 4 °C. To minimize non-specific binding of PrP to the plastic tube walls, the tubes were treated with chlorodimethylsilane (silanized tubes) before the experiment. Samples were collected at specific time points; proteins were extracted with 40 µL of a 5xSDS sample buffer at 100 °C for 10 min, and stored at −80 °C until analyzed by immunoblotting. These samples are not protease digested as the PK binds to the soils and the extraction conditions, SDS and boiling, resulting in denatured PK-sensitive prion protein. Samples (10 µL) were resolved on 12- or 15-well 12% NuPAGE bis-Tris gels (Invitrogen), transferred to PVDF membrane and probed with the anti-PrP antibody Bar 224 for PrP^CWD^. 

### 2.4. Animal Bioassay 

tgElk mice were inoculated intraperitoneally (i.p.) with 100 µL of 1% BH (from transgenic mice infected with elk prions) collected after 1 and 30 weeks incubation with quartz and Luvisolic Ae horizon. Soil/mineral samples were re-suspended in water, and pasteurized 10 min at 80 °C. Controls included the inoculation of equivalent amounts of 1% CWD-BH or uninfected BH (1%) incubated for 1 week at 4 °C. In a separate study, BH (1% tgElk-CWD) were incubated with Luvisolic LFH and Ae and Chernozemic Ah horizons for 1 and 55 weeks, the samples were re-suspended in water, pasteurized 10 min at 80 °C, and 100 µL were inoculated (i.p.) into tgElk mice. CWD-BH (1% and 0.01%) incubated for 1 week or uninfected BH (1%) were also pasteurized and inoculated as controls. In both bioassays, mice were monitored daily for the onset of clinical signs and euthanized upon confirmed clinical disease. Brains from clinically positive mice and uninfected controls were analyzed for protease-resistant PrP (PrP^res^) by immunoblotting as described above. For the proteinase digestion reactions, 50 μg total proteins were treated with 75 μg/mL of proteinase K (Life Technologies) for 45 min at 37 °C. Reactions were terminated by adding of the 40 µL of 5xSDS sample buffer and boiling at 100 °C for 10 min.

## 3. Results and Discussion

### 3.1. Decline in PrP^CWD^ Recovery with Increasing Soil/Mineral Incubation Time

**Figure 1:**
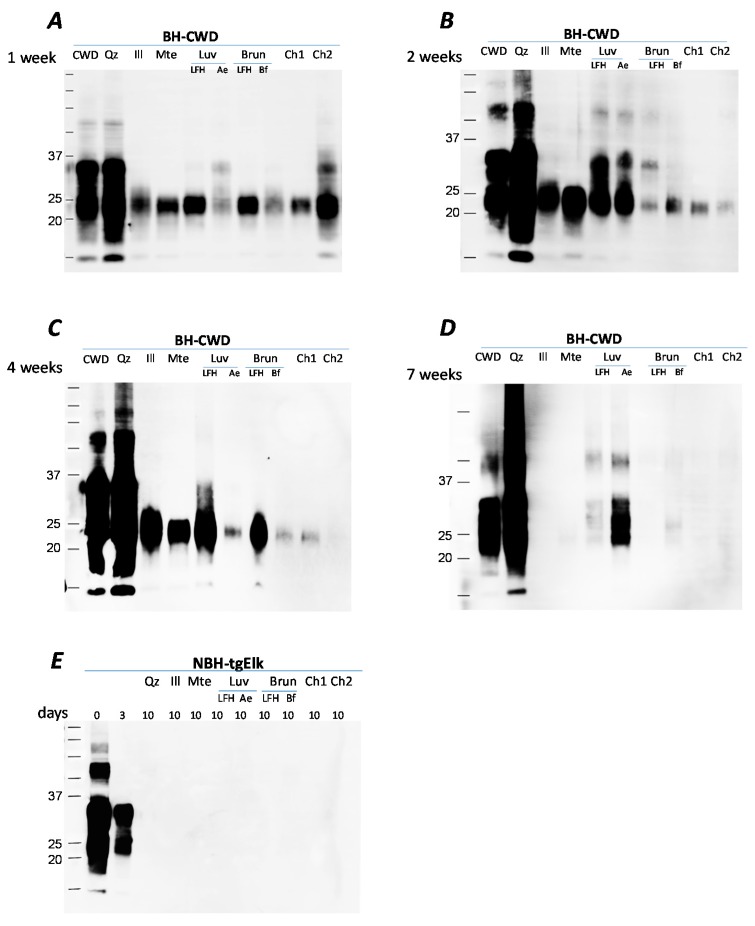
Decreasing PrP signal after incubation with soils and soil minerals for 7 weeks. Proteins were extracted after 1 (**Panel A**), 2 (**Panel B**), 4 (**Panel C**) or 7 (**Panel D**) weeks incubation with water (control), quartz, illite, Mte, Luvisolic horizons LFH or Ae, Brunisolic horizons LFH and Bf and Ah horizons from 2 Chernozems. (**Panel E**): uninfected brain homogenate (BH) incubated for 0, 3 and 10 days. Identical amounts of 1% chronic wasting disease (CWD)-infected or uninfected BH (tgElk-CWD, final concentration 0.25%) were incubated with water (control) soils or soil minerals at 4 °C in silanized tubes. Samples were taken at specified time points and analyzed by western blot using Bar224 antibody.

Long-term incubation of CWD-infected BH with illite, Mte and soil horizons results in a time-dependent decline in recovery of PrP^CWD^ (Figure 1). After one week of incubation, PrP^CWD^ signal intensity decreased for all mineral/soil combinations, except quartz, in comparison to the control (1% BH diluted in water without solid substrate) (Figure 1A). Samples were collected at specified time points over 7 weeks and were treated with SDS (diluted to a final concentration 0.14% of BH) and incubated at 100 °C, a procedure from which we have successfully recovered prions avidly bound to clay minerals [11]. After 7 weeks of incubation, PrP^CWD^ was not detectable, by immunoblot, from illite, Mte, Brunisolic LFH and both Chernozemic horizon samples, but still detected in Luvisolic horizons (plant litter LFH and eluvial Ae horizon), and weakly detected for Bf Brunisolic horizon. PrP^CWD^ was detectable in both the BH+quartz and BH control throughout the 7 weeks (Figure 1). Although the PrP signal after incubation with quartz did not decline, we observed the glycosylation pattern became smeared (Figure 1A–D). For all other minerals and soil horizons PrP desorption is characterized by truncation of the N-terminal fragment (~35kDa); this cleavage has been described previously for Mte and the fate of the cleaved amino acid residues has not been determined [11,23]. Similar experiments with uninfected brain homogenate resulted in no detection of PrP^C^ after 10 days of incubation with soils or soil minerals (Figure 1E) suggesting that the PrP detected following extended soil/soil mineral incubations is PrP^CWD^.

**Figure 2:**
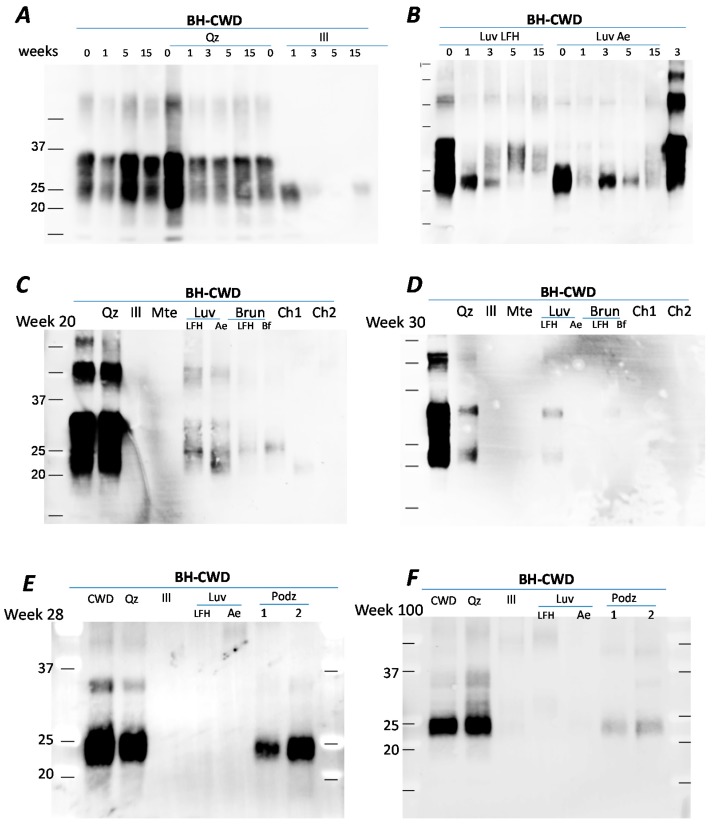
PrP extracted after incubation with soils or soil minerals. (**A**) PrP after 1, 5 or 15 weeks incubation with water (control), quartz or illite; (**B**) PrP after 1, 5 or 15 weeks incubation with Luvisolic horizons LFH or Ae; (**C**) PrP after 20 weeks incubation with quartz, illite, Mte, Luvisolic horizons LFH or Ae, Brunisolic horizons LFH and Bf and Ah horizons from 2 Chernozems; (**D**) PrP signal extracted after 30 weeks incubation; (**E**,**F**) PrP after incubation with soils and soil minerals for 28 and 100 weeks incubation with water (control), quartz, illite, Mte, Luvisolic horizons LFH and Ae, and Ae horizons from two Podzols. Identical amounts of 1% CWD-infected brain homogenate from tg33 mice ((**Panels A**–**D**); final concentration 0.25%) and tgElk mice ((**Panels E**,**F**); final concentration 0.25%) were incubated with water (control) or 15 mg/mL suspension of soils/soil minerals at 4 °C in silanized tubes. Samples were taken at specific time points and analyzed by western blot using Bar224 antibody.

We repeated the experiment for longer periods of time (up to 55 weeks) using the same minerals and soils (Qz, illite, Mte, Luvisol, Brunisol and Chernozems) and found a similar trend. After prolonged incubation of 15 weeks, PrP^CWD^ signal from BH or BH+quartz signal was stable (Figure 2A), while incubation with illite resulted in reduced PrP^CWD^ signal. After 15 weeks incubation with two Luvisolic horizons (LFH and Ae), PrP detection continued to decrease (Figure 2B) in comparison to the BH control (last band, Figure 2B). Comparison between the samples after 20 weeks of incubation revealed that PrP^CWD^ signal in BH control or BH+quartz signal were similarly intense (Figure 2C), but PrP^CWD^ was not detectable from the illite and Mte samples. Amongst the soils, the strongest PrP^CWD^ detection was from the Luvisolic horizons, followed by the Brunisols, with the lowest PrP^CWD^ recovery associated with the Chernozems. After 30 weeks incubation, PrP^CWD^ signal from BH remained the same, but was no longer detectable in any soil from this set (Figure 2D). Incubation of CWD-BH with 2 Podzol soils, sandy soils with low humus content collected in Scandinavia, showed stable PrP signal during the first 28 weeks (Figure 2E) while exhibiting a decline after 100 weeks of incubation (Figure 2F). Across the soils and time-points, recovery of PrP^CWD^ was higher from the Podzolic and Luvisolic soils, followed by the Brunisols, and finally the Chernozems. Variation in soil types and differences in their properties such as minerology, clay and humus content, are likely the main factors responsible for PrP persistence and recovery after prolonged incubation.

### 3.2. Mineral/Soil Interaction Has Little Effect on Infectivity

**Figure 3:**
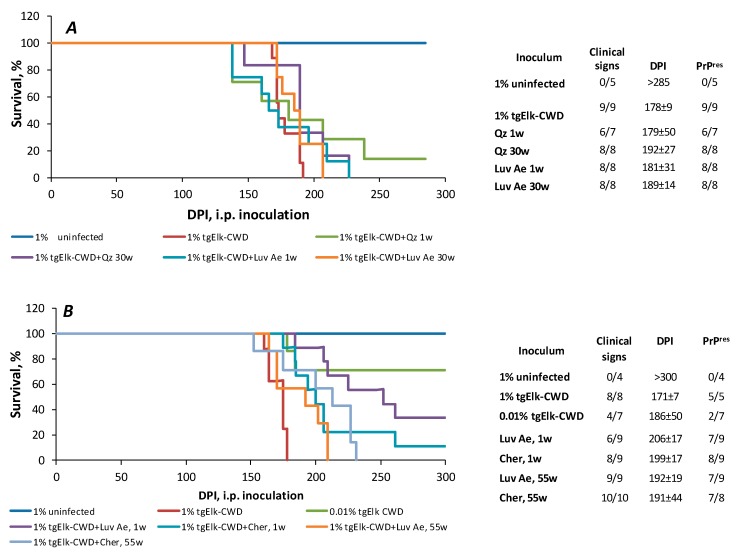
Animal bioassays. (**A**) Survival curve of tgElk mice i.p. inoculated with 1% CWD-BH incubated with quartz (Qz) and Luvisolic Ae horizon for 1 and 30 weeks; (**B**) Survival curve of tgElk mice i.p. inoculated with 1% CWD-BH incubated with Luvisolic Ae and Chernozemic Ah horizons for 1 week and 55 weeks.

Animal bioassay experiments of Luvisolic Ae horizon (1, 30 and 55 week incubation) and Chernozemic Ah horizon (1 and 55 week incubation) were performed to determine the effect of prolonged soil mineral/soil interaction on infectivity (Figure 3). Infectious brain homogenate (no soil mineral) and quartz sand (little decline in PrP^CWD^ with prolonged incubation) were included as controls. Extended incubation of CWD infectivity with soil horizons had little effect on infectivity. Thirty week and 55 week incubation of Luvisolic Ae horizon produced similar infectivity to 1 week Luvisolic Ae. Similarly, Chernozemic Ah horizon bound to CWD for 55 weeks resulted in infectivity levels similar to the 1 week soil/PrP^CWD^ interaction (Figure 3A).

Similar to the previous experiments, the amount of detectable PrP recovered from the inoculums for this bioassay differed with PrP being detectable for 1% and 0.01% CWD-BH control as well as for 1 week old BH+Luvisolic Ae horizon, while the CWD-BH/soil samples incubated 55 weeks were not detectable by immunoblotting (Supplementary Figure S2). All control animals inoculated with 1% BH (unbound PrP, control) exhibited clinical signs by 171 ± 7 dpi (Figure 3B). While four out of seven mice inoculated with 0.01% BH (elk-CWD) showed clinical signs (186 ± 50 dpi), most mice inoculated with soil samples (CWD-BH pre-incubated with soils) exhibited clinical signs and PrP^res^ accumulation in brain. There is no significant difference between inocula in incubation periods; mice inoculated with soil samples have an incubation period of appx. 200 dpi with a wide standard deviation. Exact incubation periods for each inoculum and PrP accumulation in harvested brains are shown in Figure 3, Supplementary Figures S3 and S4, respectively.

### 3.3. Declining Prion Recovery Yet Infectivity Remains

To summarize, the difference between inocula, as detected by immunoblotting (no signal after prolonged incubation with soils) did not correlate with the incubation period. The bioassay did not suggest a decrease in infectivity of soil-bound prions or PrP degradation. There are several possible explanations for the observed decline in immunoblot PrP^CWD^ signal upon prolonged incubation with soils and minerals, including adsorption to soil, adsorption to the tube, or inactivation of the antibody epitope. To minimize adsorption to Eppendorf tubes, we coated them with chlorodimethylsilane. Note that, the BH alone serves as a sorption control; if adsorption was a significant factor, we would observe decreasing levels of PrP^CWD^ for the BH control, however it was stable over 30 weeks (Figure 2). To determine whether the antibody epitope was removed we used mAb D15.15 (epitope 176–178), SAF83 (epitope 126–164 excl. 142–160) and 8G8 (epitope 97–102), in addition to the mAb Bar224 (epitope 141–151). We obtained similar results for all antibodies where PrP signal remained stable for the BH control sample and decline or disappeared in samples with soils (Supplementary Figure S1). The data suggest the most probable explanation that the PrP^CWD^ adsorption to soil might increase with time because it depends on competitive sorption effects of the brain homogenate matrix where PrP^CWD^ initially competes for sorption sites with thousands of other constituents. Over time, more surface area in soils may become available due to degradation, reorientation, or conformational changes of bound molecules, allowing more stable PrP^CWD^ to adsorb. We previously demonstrated that prion binding to some soils enhances infectivity [17]. It is, therefore, possible that PrP^CWD^ levels declined with extended soil incubations but infectivity levels are enhanced. This possibility does not alter our conclusion that declining ability to detect PrP^CWD^ when bound to soils for extended times does not result in a concomitant decline in CWD infectivity.

Clearly, the binding of whole soils and soil minerals is complex and the interaction can be exceedingly avid. Even short incubation times with the soil mineral montmorillonite require extreme conditions (10% SDS, 100 °C) to facilitate recovery of PrP^CWD^. The decline in PrP^CWD^ recovery with increasing incubation time varied with soil type. Soils from prairie regions (montmorillonite mineralogy and high humus content) show the highest ability to bind PrP^CWD^, where PrP^CWD^ was not recoverable after 4 weeks of incubation with those soils. Prion incubation with soils from boreal regions (illite/quartz mineralogy, low humus content) result in more stable recovery of PrP^CWD^ signal, recoverable up to 28 weeks from Podzols and up to 20 weeks from Luvisols and Brunisols.

Sensitive detection of PrP^CWD^ prions from environmental samples would greatly simplify disease monitoring. The declining ability with time to detect CWD prions bound to soils significantly complicates such an approach. Alternative methods of extractions of prions from soil are required, and extractions that retain PrP^CWD^ structure would facilitate sensitive prion amplification technologies (e.g., PMCA, RT-QuIC). The ability to in vitro amplify soil-bound prions varies significantly with soil type; it is significantly reduced when bound to clay soil and soil organic matter compared to unbound and sand-bound PrP^Sc^ [26]. Although in vitro amplification technologies are the most sensitive and promising method to detect soil-bound prions, and were used to detect PrP^CWD^ in naturally contaminated water and soil samples [12], PrP^CWD^ recovery efficiency is not sufficient to detect infectious prions in a wide range of environmental samples. 

## 4. Conclusions

The binding of prions to soil minerals and other soil constituents impacts PrP^CWD^ recovery. During extended incubation with soils, PrP signal on immunoblots continuously declined until it was no longer detectable after 25 weeks in soils with loamy-clay texture and Mte minerology. PrP^CWD^ infectivity did not, however, decrease after 30 weeks incubation with quartz and the Luvisolic Ae soil horizon. At 55 weeks incubation in Chernozem and Luvisol, CWD-BH remained infectious. We studied a wide variety of soil types (from prairie, mountain and boreal regions) and showed decreased PrP^CWD^ signal recovery (as measured by immunoblotting) with retention of infectivity. The decrease in PrP^CWD^ recovery was particularly dramatic in soils from the prairie region. Regardless of soil minerology, texture and humus content, detection of PrP^CWD^ in environmental soil samples is a challenge after long-term incubation. These findings provide important information on the behavior of prions in natural environments, but complicate analysis of environmental samples.

## Supplementary Materials

The following are available online at https://www.mdpi.com/2076-0817/9/4/311/s1, Figure S1: Decreasing of PrP signal extracted after incubation with soils and soil minerals for 5 weeks; Figure S2: Inoculum for animal bioassay with tgElk mice i.p. inoculated with 1% CWD-BH incubated with Luvisolic Ae, LFH and Chernozemic Ah horizons for 1 and 55 weeks; Figure S3: Accumulation of PrP^res^ in brains harvested from mice with clinical signs. Numbers represent days after inoculation when animal was harvested; Figure S4: Accumulation of PrP^res^ in brains harvested from mice with clinical signal and at the end of experiment (without clinical signals).
