# Supplementary material for: Long-Term Incubation PrPCWD with Soils Affects Prion Recovery but Not Infectivity
Source: Pathogens. 2020 Apr 23;9(4):311. doi: 10.3390/pathogens9040311 (PMC7238116; doi:10.3390/pathogens9040311)
Supplement: Supplementary file 1 [file pathogens-09-00311-s001.pdf]

## Supplementary information: Figures S1-S4

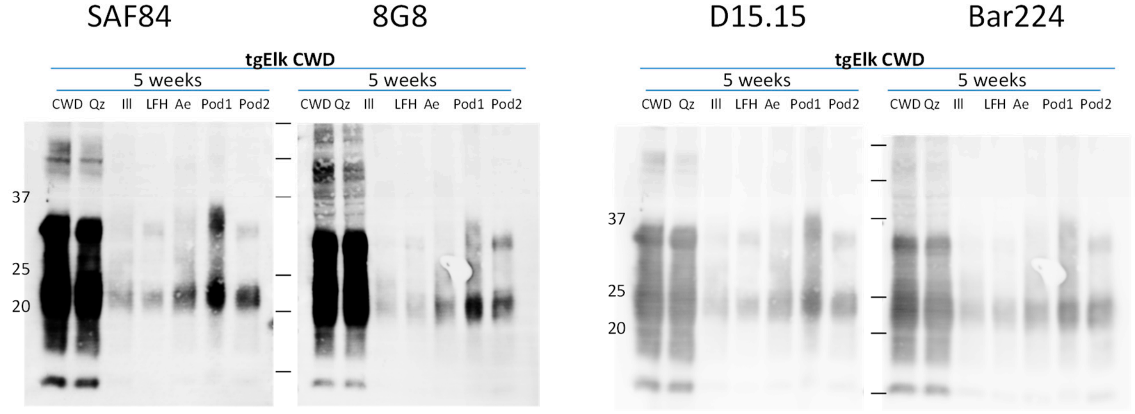


**Figure S1.** Decreasing of PrP signal extracted after incubation with soils and soil minerals for 5 weeks.

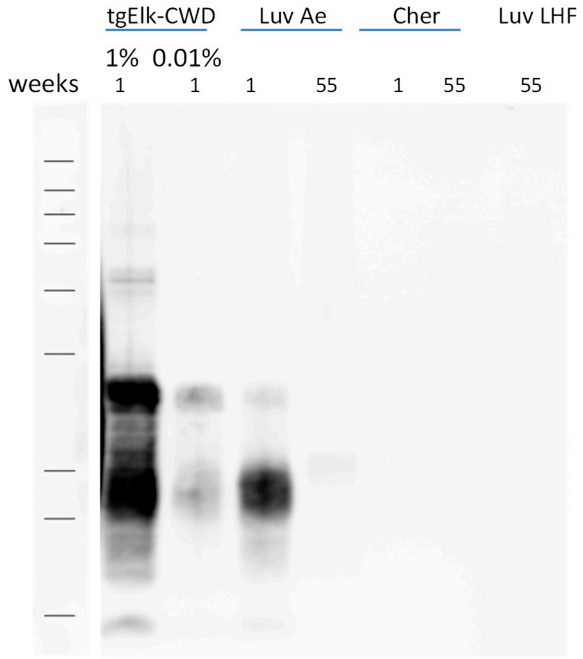


**Figure S2.** Inoculum for animal bioassay with tgElk mice i.p. inoculated with 1% CWD-BH incubated with Luvisolic Ae, LFH and Chernozemic Ah horizons for 1 week and 55 weeks.

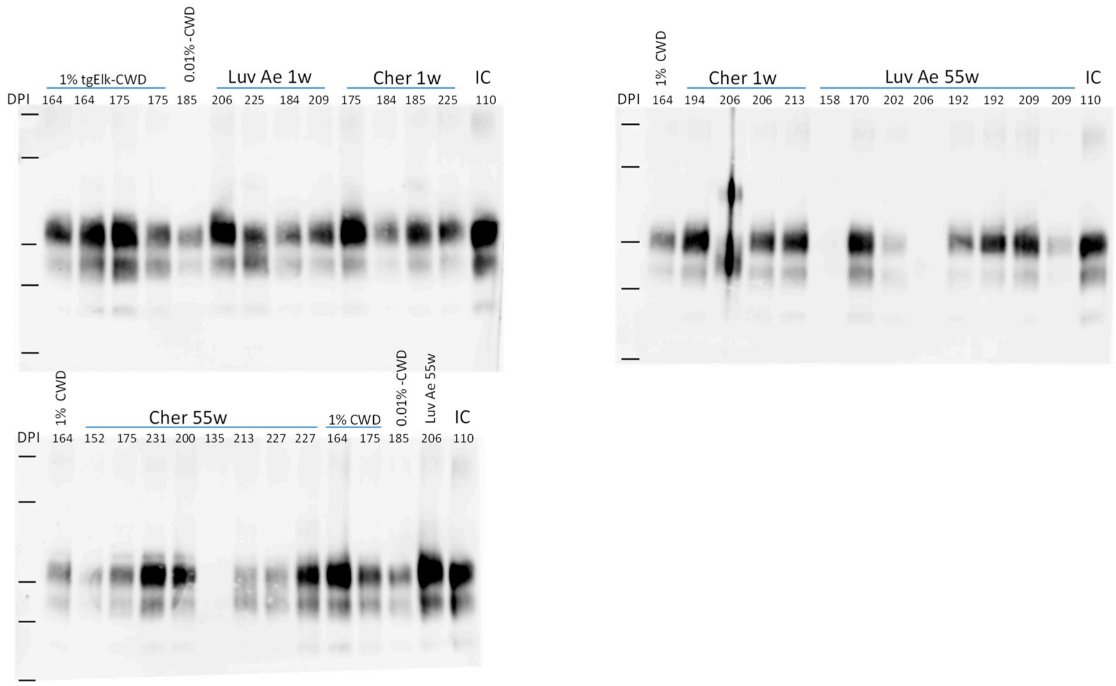


**Figure S3.** Accumulation of PrP[res] in brains harvested from mice with clinical signs. Numbers represent days after inoculation when animal was harvested.

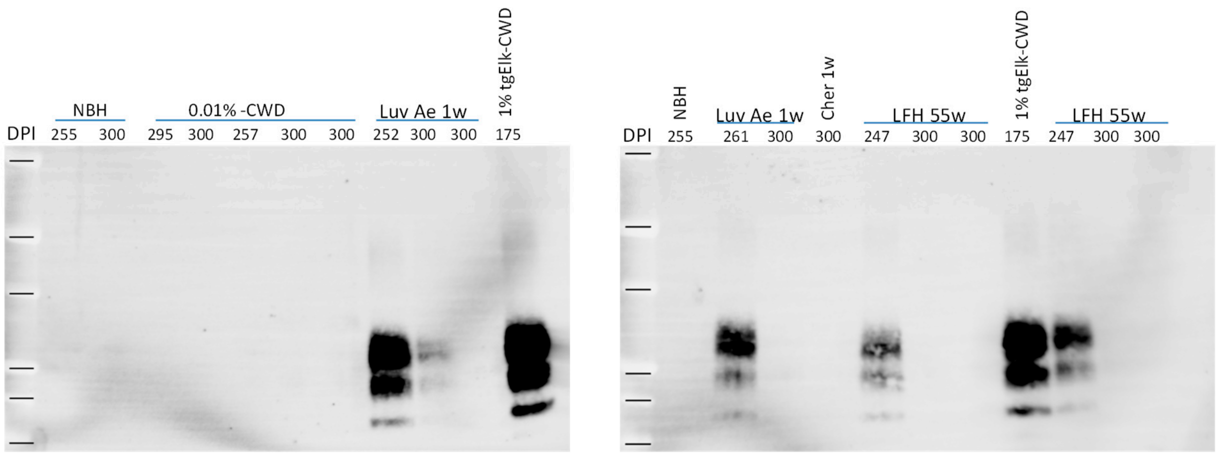


**Figure S4.** Accumulation of PrP[res] in brains harvested from mice with clinical signal and at the end of experiment (without clinical signs).
